# Computed tomography-derived area and density of pectoralis muscle associated disease severity and longitudinal changes in chronic obstructive pulmonary disease: a case control study

**DOI:** 10.1186/s12931-019-1191-y

**Published:** 2019-10-21

**Authors:** So Hyeon Bak, Sung Ok Kwon, Seon-Sook Han, Woo Jin Kim

**Affiliations:** 1 Department of Radiology, Kangwon National University Hospital, Kangwon National University School of Medicine, Chuncheon, Republic of Korea; 2 Biomedical Research Institute, Kangwon National University Hospital, Chuncheon, Republic of Korea; 3 Department of Internal Medicine and Environmental Health Center, School of Medicine, Kangwon National University, 1 Kangwondaehak-gil, Chuncheon, Gangwon-do 24341 Republic of Korea

**Keywords:** COPD, Pectoralis muscle, Imaging

## Abstract

**Background:**

Muscle wasting is associated with prognosis in patients with chronic obstructive pulmonary disease (COPD). The cross-sectional area of skeletal muscles on computed tomography (CT) could serve as a method to evaluate body composition. The present study aimed to determine the ability of CT-derived pectoralis muscle area (PMA) and pectoralis muscle density (PMD) to determine the severity of COPD and change in longitudinal pulmonary function in patients with COPD.

**Methods:**

A total of 293 participants were enrolled in this study, a whom 222 had undergone at least two spirometry measurements within 3 years after baseline data acquisition. PMA and PMD were measured from a single axial slice of chest CT above the aortic arch at baseline. The emphysema index and bronchial wall thickness were quantitatively assessed in all scans. The generalized linear model was used to determine the correlation between PMA and PMD measurements and pulmonary function.

**Results:**

PMA and PMD were significantly associated with baseline lung function and the severity of emphysema (*P* < 0.05). Patients with the lowest PMA and PMD exhibited significantly more severe airflow obstruction (*β* = − 0.06; 95% confidence interval: − 0.09 to − 0.03]. PMA was statistically associated with COPD assessment test (CAT) score (*P* = 0.033). However, PMD did not exhibit statistically significant correlation with either CAT scores or modified Medical Research Council scores (*P* > 0.05). Furthermore, neither PMA nor PMD were associated with changes in forced expiratory volume in 1 s over a 3-year periods.

**Conclusions:**

CT-derived features of the pectoralis muscle may be helpful in predicting disease severity in patients with COPD, but are not necessarily associated with longitudinal changes in lung function.

## Background

Chronic obstructive pulmonary disease (COPD), characterized by a progressive decline in airflow, is the leading cause of death worldwide and is related to systemic manifestations and comorbid conditions, including ischemic heart disease, osteoporosis, diabetes and muscle wasting [1–3]. Altered body composition is commonly associated with COPD, and the prevalence of muscle wasting is 20% in those with COPD [4, 5]. Muscle wasting leads to decreased skeletal muscle function and exercise capacity, enhanced energy expenditure, and compromised overall health status. Collectively, these factors contribute to increased mortality in patients with COPD [6]. Considering the correlation between body mass index (BMI) and mortality rate, independent of lung function, body weight and BMI have been conventionally used to assess muscle wasting in patients with COPD [7, 8]. However, BMI can underestimate muscle wasting because the decline in skeletal muscle mass may not always be accompanied by a corresponding loss in fat mass [5, 9, 10]. Hence, BMI does not accurately represent muscle wasting.

Chest computed tomography (CT) is widely used to assess disease severity, exclude other underlying diseases and evaluate extrapulmonary manifestations, including muscle wasting, in routine clinical practice. The CT cross-sectional area of the pectoralis, erector spinae, and midthigh muscles has been reported to be correlated with fat-free mass, symptoms, disease severity, and prognosis in patients with COPD [11–13]. McDonald et al. recently reported that CT-derived pectoralis muscle area (PMA) is a crucial predictor of COPD-related outcomes, and that smaller PMA is associated with severe airflow limitation, lower quality of life, and decreased exercise capacity [12]. CT-derived PMA is a reproducible measure of muscle mass, which can be obtained without additional radiation exposure or cost [14]. Moreover, CT can identify sex differences in body composition including breast tissue in women [4]. In addition to cross-sectional muscle area, CT-derived measurements of muscle density are an indicator of muscle quality. Low muscle density reflects lipid-rich skeletal muscle, which is correlated with increased disability and decreased physical function [15]. Skeletal muscle dysfunction may affect both ventilator and limb muscles in patient with COPD. However, dysfunction in the limb muscles is more severe than in the ventilator muscles due to positive adaptation (training-like effect) of the respiratory muscles [16]. Weakness in the quadriceps muscles is associated with modified Medical Research Council (mMRC) and BODE (body mass index, airflow obstruction, dyspnea, exercise capacity) [17]. Furthermore, information obtained from CT scans of the pectoralis muscle could be clinically valuable if it can be associated with the severity or prognosis of COPD. Therefore, we hypothesized that changes in the area and quality of the pectoralis muscle could lead to longitudinal changes in lung function. To the best of our knowledge, no study has investigated the effect of pectoralis muscle density (PMD) or established correlations between PMA and PMD with changes in longitudinal pulmonary function in those with COPD. Hence, this study aimed to investigate the impact of CT-derived features of the pectoralis muscle including PMA and PMD on the manifestation of COPD and changes in longitudinal pulmonary function in patients with COPD.

## Material and methods

### Study population

**Fig. 1:**
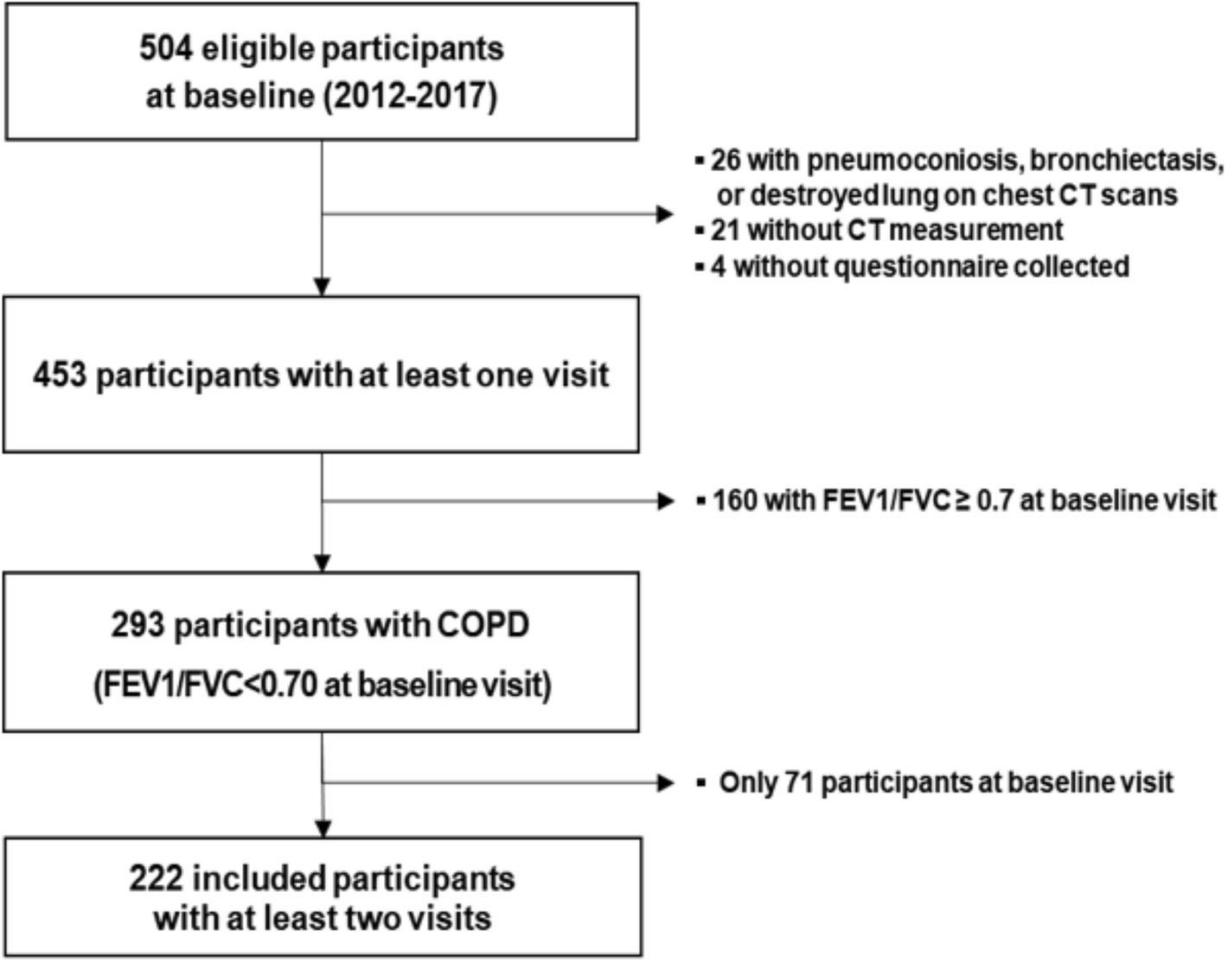
The flowchart for study participants

Initially, 504 participants were selected from a Korean cohort [the Chronic Obstructive Pulmonary Disease in Dusty Areas (CODA) cohort] from 2012 to 2017, which aimed to assess the clinical outcomes of participants residing near cement factories. All enrolled participants were assessed by medical interview, physical examination, spirometry, laboratory tests and chest CT scan at the start of the study. The study design is detailed elsewhere [18]. COPD is diagnosed when patients have postbronchodilator forced expiratory volume in 1 s (FEV_1_)/forced vital capacity (FVC) < 0.7 at baseline [19]. Individuals with an FEV_1_/FVC ratio > 0.7 (*n* = 160), no questionnaire data (*n* = 4), severe lung parenchymal distortion due to pneumoconiosis, bronchiectasis and/or pulmonary tuberculosis (*n* = 26) and the absence or severe asymmetry of the pectoralis muscle due to nerve denervation or disability (*n* = 21) were excluded (*n* = 211). Ultimately, 293 participants were enrolled of whom 222 underwent at least two postbronchodilator spirometry measurements within 3 years after baseline (average 3.5 visits per person) data acquisition and comprised the sample for the longitudinal analysis: 1) 35 subjects (15.8%) who had two spirometry measurements, 2) 37 subjects (16.7%) who had three spirometry measurements, 3) 150 subjects (67.5%) who had four spirometry measurements (Fig. 1). This study was approved by the Institutional Review Board of the Kangwon National University Hospital (approval #2012–06-007), and all participants provided written informed consent.

### Clinical variables and pulmonary function

The interview questionnaire comprised demographic data, medical history, environmental exposure and respiratory symptoms at baseline. While dyspnea was assessed using the mMRC scoring system, quality of life was assessed using the patients-reported COPD assessment test (CAT).

Spirometry was performed yearly from enrollment using the Easy One Kit (NDD Medizintechnik AG, Zurich, Switzerland)), before and after inhalation of 400-μg salbutamol. Notably, all pulmonary function tests conformed to the guidelines of the American Thoracic Society/European Respiratory Society [20].

### CT acquisition and image analysis

All patients underwent volumetric, thin-section, chest CT at full inspiration and expiration in the supine position. CT images were acuqired using a first-generation, dual-source CT scanner (Somatom Definition; Siemens Healthcare, Forchheim, Germany) in a caudocranial direction using the following parameters: 140 kVp, 100 mA, 0.9–1 beam pitch and slice thickness 0.6 mm and 3 mm. CT data were reconstructed using a soft convolution kernel (B30f). The pectoralis muscle was evaluated using mediastinal window images [width, 400 Hounsfield units (HU); level, 20 HU].

**Fig. 2:**
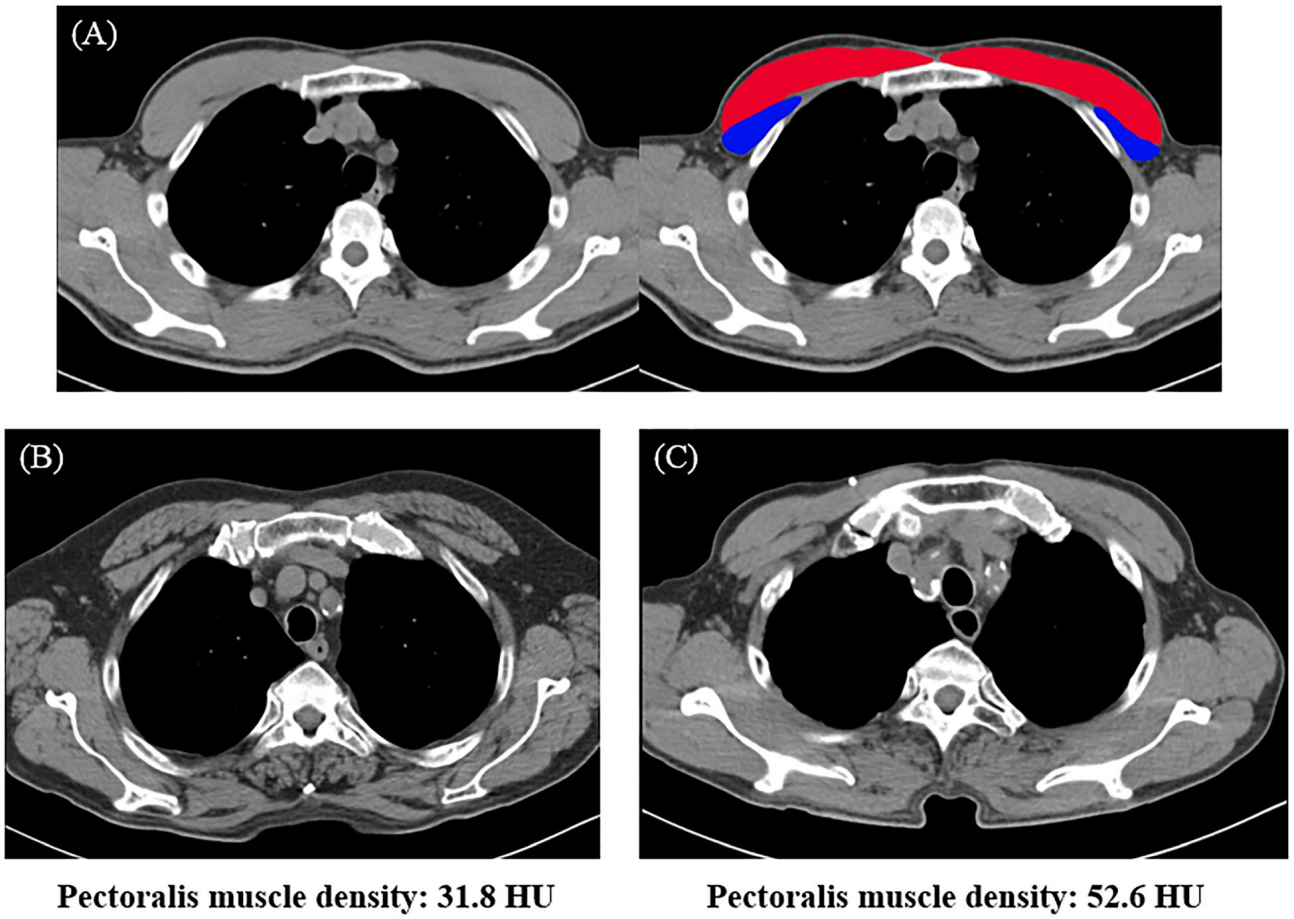
Computed tomography (CT) scans were used to assess the pectoralis muscle area and density. **a**, the CT axial slice above the aortic arch level shows the pectoralis muscle segmentation (red, pectoralis major muscle; blue, pectoralis minor muscle). **b** and **c**, differences between pectoralis muscle densities are depicted in CT images

PMA and PMD were measured on a single axial slice of the chest CT scan above the aortic arch at baseline CT (Fig. 2) [12]. The pectoralis muscle was segmented by drawing a region of interest (ROI) that traced along the edge of the right and left pectoralis major and minor muscles; COPD severity was blinded during the analyses. PMA (in cm^2^) was evaluated as the sum of the left and right pectoralis major and minor muscles. PMD (in HU) was defined as the mean attenuation within the ROI that segmented the pectoralis muscle.

Using in-house software, whole-lung images were automatically extracted from the chest wall, mediastinum and large airways to quantitatively assess emphysema and bronchial wall thickness. Subsequently, the attenuation coefficients of pixels in these images were measured. The emphysema index (EI) was defined as the volume fraction (%) of the lung below − 950 HU at full inspiration [21]. Airway dimensions, including wall area (WA), lumen area, and WA% [defined as WA/(WA + lumen area) × 100], were measured near the origin of the right apical and left apicoposterior segmental bronchi [22]. Furthermore, WA% was used to assess airway thicknesses and the mean values of segmental bronchi in the statistical analyses; these CT measurements were performed on each participant at baseline.

### Statistical analyses

To evaluate the effect PMA and PMD on symptoms and lung function in COPD, the general linear model for baseline data analyses and the generalized linear mixed model for longitudinal data analyses were used. Participants were categorized into three tertiles according to PMA (in cm^2^) or PMD (in HU): tertile 1 (lowest), tertile 2, and tertile 3 (highest). Effect estimates were reported as the difference in pulmonary functions parameters, with participants in the third tertile of PMA and PMD as the reference or for each one-unit increment in PMA and PMD. Moreover, the effects on COPD manifestation were estimated by combining PMA and PMD scores. For the latter analyses, both PMA and PMD were categorized into high (tertile 3) and low (tertile 1– tertile 2) groups and then cross-classified, yielding the following four categories: (i) low PMA + low PMD; (ii) low PMA + high PMD; (iii) high PMA + low PMD; and (iv) high PMA + high PMD (reference group). Furthermore, multivariable regression models were used to adjust for confounders of age, sex, BMI, smoking status, pack-years, and history of acute exacerbation. A subgroup analysis was conducted on males.

In the longitudinal data analyses, the adjusted difference in annual changes in lung function (FEV_1_) over time in association with CT-derived features of the pectoralis muscle (i.e., PMA and PMD) were assessed by framing a model that compared CT features of each visit with the baseline CT. The model was adjusted for baseline age, sex, height, education level, COPD medication use, smoking status, and pack-years. The coefficient of the interaction term was used to evaluate the additional decline in annual lung function percentages over time, for each one-unit increment in CT-derived features of the pectoralis muscles (i.e., PMA and PMD).

*P* < 0.05 was considered to be statistically significant. All statistical analyses were performed using SAS version 9.4 (SAS Institute, Cary, NC, USA).

## Results

### Patient characteristics

**Fig. 3:**
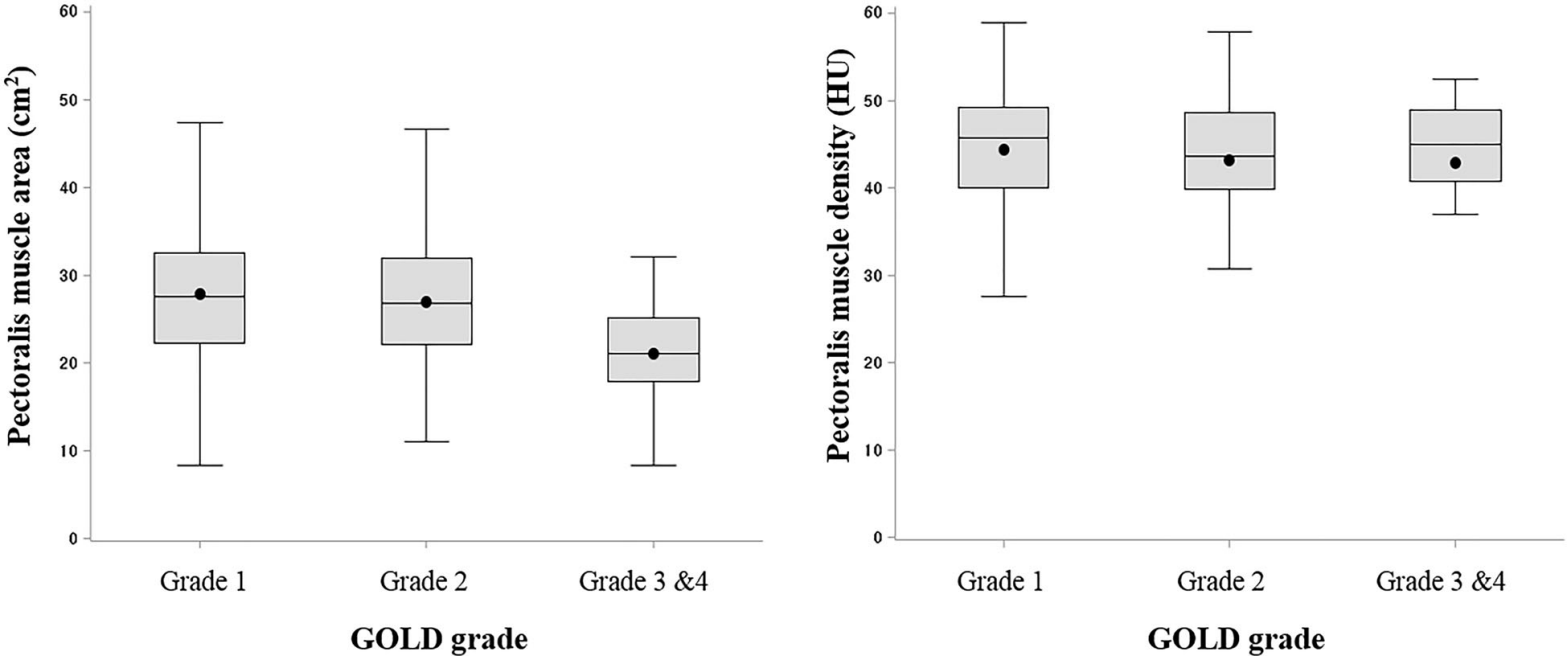
Distribution of pectoralis muscle area (in cm^2^) and density (in HU) by GOLD grade in cross-sectional analysis. *Definition of abbreviations*: GOLD, Global Initiative for Chronic Obstructive Lung Disease

A total of 293 patients (236 male, 57 female; mean age, 72.30 ± 6.97 years; range, 44.0–96.0 years) underwent measurement of BMI (23.12 ± 3.10 kg/m^2^), mMRC score (1.45 ± 1.14), CAT score (17.05 ± 9.64) and FEV_1_ (80.02% ± 19.73%). In addition, mean EI (7.69% ± 7.25%), WA% (69.32 ± 4.97), PMA (27.01 ± 8.11 cm^2^) and PMD (43.85 ± 7.51 HU) were assessed for all participants. The distribution of PMA (in cm^2^) and PMD (in HU) according to Global Initiative for Chronic Obstructive Lung Disease (GOLD) grade in cross-sectional analysis is shown in Fig. 3. Pearson correlation coefficient between PMA and PMD was 0.30 (*P* < 0.001). Baseline characteristics and sex differences in the study cohort are summarized in Table 1.

**Table 1:** The baseline characteristics of patients with COPD in the CODA cohort (*n* = 293)

	Total (*n* = 293)	Male (*n* = 236)	Female (*n* = 57)
Gender^a^			
Male	236 (80.6)		
Female	57 (19.5)		
Age	72.30 ± 6.97	71.95 ± 7.10	73.75 ± 6.27
Smoking^a^			
Never-smoker	77 (26.3)	23 (9.8)	54 (94.7)
Former-smoker	140 (47.8)	138 (58.5)	2 (3.5)
Current-smoker	76 (25.9)	75 (31.8)	1 (1.8)
Pack-years	17.53 ± 23.50	23.54 ± 24.11	1.33 ± 10.74
Height (cm)	160.27 ± 8.56	163.35 ± 5.91	147.53 ± 5.51
Weight (kg)	59.54 ± 10.11	61.42 ± 9.71	51.75 ± 7.78
BMI (kg/m^2^)	23.12 ± 3.10	22.97 ± 3.13	23.74 ± 2.92
< 23.0^a^	141 (48.1)	118 (50.0)	23 (40.4)
23.0–24.9^a^	66 (22.5)	52 (22.0)	14 (24.6)
≥ 25.0^a^	86 (29.4)	66 (28.0)	20 (35.1)
GOLD grade^a^			
Grade 1	151 (51.5)	118 (50.0)	33 (57.9)
Grade 2	118 (40.3)	96 (40.7)	22 (38.6)
Grade 3	21 (7.2)	19 (8.1)	2 (3.5)
Grade 4	3 (1.0)	3 (1.3)	0 (0.0)
Pulmonary function			
FVC, L	3.10 ± 0.81	3.31 ± 0.72	2.24 ± 0.54
FVC, % predicted	98.33 ± 19.24	96.89 ± 18.54	104.26 ± 21.09
FEV_1_, L	1.84 ± 0.56	1.95 ± 0.55	1.36 ± 0.32
FEV_1_, % predicted	80.02 ± 19.73	78.66 ± 19.31	85.65 ± 20.64
FEV_1_/FVC	0.59 ± 0.08	0.58 ± 0.09	0.61 ± 0.07
mMRC	1.45 ± 1.14	1.37 ± 1.10	1.81 ± 1.25
Acute exacerbation^a^			
None	275 (93.9)	224 (94.9)	51 (89.5)
Yes1	18 (6.1)	12 (5.1)	6 (10.5)
≥ 2 moderate acute exacerbation	10 (3.4)	7 (3.0)	3 (5.3)
≥ 1 severe acute exacerbation	13 (4.4)	9 (3.8)	4 (7.0)
CAT score	17.05 ± 9.64	16.45 ± 9.53	19.54 ± 9.79
CT-features			
Mean wall area %	69.32 ± 4.97	68.63 ± 4.71	72.16 ± 5.06
Emphysema index	7.69 ± 7.25	8.85 ± 7.48	2.91 ± 3.23
Pectoralis muscle area (cm^2^)	27.01 ± 8.11	28.80 ± 7.72	19.57 ± 4.84
Pectoralis muscle density (HU)	43.85 ± 7.51	45.44 ± 6.06	37.27 ± 9.22

Data are mean ± standard deviation (SD) unless indicated otherwise

*Abbreviations*: *BMI* body mass index, *CAT* chronic obstructive pulmonary disease assessment test, *CODA cohort* chronic obstructive pulmonary disease in dust areas cohort, *COPD* chronic obstructive pulmonary disease, *CT* computed tomography, *FEV*_*1*_ forced expiratory volume in 1 s, *FVC* forced vital capacity, *GOLD* Global Initiative for Chronic Obstructive Lung Disease, *mMRC* modified Medical Research Council, *PMA* pectoralis muscle area, *PMD* pectoralis muscle density, *HU* Hounsfield unit

^a^Data are number and data in parentheses are percentages

### Relationship between PMA and COPD manifestation

After categorizing patients with COPD (*n* = 293) into three groups based on PMA (in cm^2^), each group was analyzed and compared with the highest PMA group after adjustments for age, sex, smoking, pack-years, BMI, and history of acute exacerbation (Table 2). The mean PMA values for tertiles 1, 2 and 3 were 19.34, 26.63 and 34.20 cm^2^, respectively. The group with the smallest PMA exhibited significantly lower FEV_1_ (*P* < 0.001), lower predicted FEV_1_ (*P* < 0.001), lower predicted FVC (*P* = 0.026), and severe airflow obstruction (*P* < 0.001). A 10 cm^2^ increase in PMA was significantly associated with a significant increase in 0.21 L FEV_1_ [95% confidence interval (CI), 0.12–0.30; *P* < 0.001]. Although greater PMA was associated with lower CAT scores (*P* = 0.033), no statistically significant difference was noted in mMRC (*P* = 0.358). Compared with CT measurements related to COPD, smllar PMA was significantly associated with a higher EI (*P* = 0.001), with no difference in WA% (*P* = 0.915). The subgroup analysis of males yielded similar results, except for CAT scores (Additional file 1: Table S1).

**Table 2:** The pulmonary outcome as determined by the pectoralis muscle area (PMA, in cm^2^) in a cross-sectional analysis

	Adjusted mean	Adjusted difference in mean relative to tertile 3				
	Tertile 3 (highest)^a^	Tertile 2^b^	Tertile 1 (lowest)^b^	*P*-trend	Continuous *β* (95% CI), per 10 cm^2^ increase^b^	*P*
*N*	98	98	97			
PMA (cm^2^), median (min–max)	34.20 (29.83–56.42)	26.63 (23.44 to 29.82)	19.34 (8.31–23.44)			
FVC, L	2.94 ± 0.12	−0.11 (−0.30 to 0.07)	−0.25 (− 0.48 to − 0.02)	0.036	0.17 (0.04 to 0.29)	0.008
FVC, % predicted	105.18 ± 3.57	−2.61 (−8.25 to 3.04)	−8.18 (−15.26 to −1.10)	0.026	4.47 (0.73 to 8.21)	0.019
FEV_1_, L	1.88 ± 0.08	−0.22 (− 0.36 to − 0.09)	− 0.33 (− 0.50 to − 0.16)	< 0.001	0.21 (0.12 to 0.30)	< 0.001
FEV_1_, % predicted	90.65 ± 3.59	−8.04 (−13.72 to −2.36)	−13.82 (− 20.95 to − 6.69)	< 0.001	8.02 (4.27 to 11.77)	< 0.001
FEV_1_/FVC	0.64 ± 0.02	− 0.04 (− 0.07 to − 0.02)	− 0.06 (− 0.09 to − 0.03)	< 0.001	0.04 (0.02 to 0.05)	< 0.001
mMRC	2.04 ± 0.20	− 0.13 (− 0.44 to 0.19)	0.21 (− 0.18 to 0.61)	0.358	− 0.14 (− 0.35 to 0.07)	0.200
CAT score	21.80 ± 1.69	− 0.68 (− 3.35 to 1.99)	3.97 (0.62 to 7.33)	0.033	−1.49 (−3.28 to 0.31)	0.104
Emphysema index	4.14 ± 1.17	1.36 (− 0.49 to 3.22)	4.14 (1.80 to 6.49)	0.001	−2.58 (−3.82 to − 1.34)	< 0.001
Mean wall area %	70.39 ± 0.90	0.21 (− 1.21 to 1.64)	0.08 (−1.73 to 1.89)	0.915	−0.28 (− 1.24 to 0.68)	0.563

The model was adjusted for age, gender, smoking, pack-years, BMI and history of acute exaacerbation

Each pectoralis muscle subclass was characterised in tertiles (highest tertile as reference)

*Abbreviations*: *CAT* chronic obstructive pulmonary disease assessment test, *FEV*_*1*_ forced expiratory volume in 1 s, *FVC* forced vital capacity, *mMRC* modified Medical Research Council, *PMA* pectoralis muscle area

^a^Mean ± SEM (standard error of the mean)

^b^*β*; 95% CI in parentheses (all such values)

### Relationship between PMD and COPD manifestation

After categorizing patients with COPD (*n* = 293) into three groups based on PMD (in HU), the group with the highest PMD was selected as the reference and compared with all other groups to assess pulmonary function, after adjustments for age, sex, smoking, pack-years, BMI, and history of acute exacerbation (Table 3). The mean PMD values for tertiles 1, 2 and 3 were 37.60, 44.90 and 50.66 HU, respectively. Patients with lower PMD were statistically associated with lower predicted FEV_1_ (*P* = 0.001), lower predicted FVC (*P* = 0.031), and severe airflow obstruction (*P* = 0.018). A 10 HU increase in PMD was significantly correlated with an 8.56% increase in the estimated FEV_1_ (95% CI, 5.12–12.00; *P* < 0.001). Nevertheless, PMD did not demonstrate statistically significant correlation with mMRC or CAT scores (*P* = 0.431 and *P* = 0.247, respectively). When correlating CT measurements with COPD, lower PMD was correlated with a higher EI (*P* = 0.007), with no difference in WA% (*P* = 0.413). Subgroup analysis of the males yielded similar results (Additional file 1: Table S2).

**Table 3:** The pulmonary outcome as determined by the pectoralis muscle density (PMD, in HU) in a cross-sectional analysis

	Adjusted mean	Adjusted difference in mean relative to tertile 3				
	Tertile 3 (highest)^a^	Tertile 2^b^	Tertile 1 (lowest)^b^	*P*_trend_	Continuous *β* (95% CI), per 10 HU increase^b^	*P*
N	98	98	97			
PMD, median (min-max)	50.66 (47.80 to 58.88)	44.90 (41.65 to 47.73)	37.60 (10.33–41.55)			
FVC, L	2.94 ± 0.12	− 0.05 (− 0.23 to 0.14)	− 0.08 (− 0.28 to 0.13)	0.462	0.07 (− 0.04 to 0.19)	0.207
FVC, % predicted	105.18 ± 3.57	−3.7 (−9.24 to 1.85)	−6.82 (− 13.05 to −0.59)	0.031	4.64 (1.18 to 8.10)	0.009
FEV_1_, L	1.88 ± 0.08	− 0.09 (− 0.22 to 0.05)	− 0.14 (− 0.29 to 0.01)	0.063	0.14 (0.06 to 0.23)	0.001
FEV_1_, % predicted	90.65 ± 3.59	−5.47 (−11.10 to 0.15)	− 10.28 (− 16.60 to − 3.97)	0.001	8.56 (5.12 to 12.00)	< 0.001
FEV_1_/FVC	0.64 ± 0.02	− 0.02 (− 0.04 to 0.01)	− 0.03 (− 0.06 to − 0.01)	0.018	0.04 (0.02 to 0.05)	< 0.001
mMRC	2.04 ± 0.20	0.1 (− 0.21 to 0.41)	0.14 (− 0.21 to 0.49)	0.431	−0.17 (− 0.37 to − 0.02)	0.077
CAT score	21.80 ± 1.69	0.81 (−1.86 to 3.47)	1.76 (−1.23 to 4.75)	0.247	−1.82 (−3.48 to −0.16)	0.032
Emphysema index	4.14 ± 1.17	2.05 (0.22 to 3.88)	2.80 (0.73 to 4.86)	0.007	−2.50 (−3.62 to −1.38)	< 0.001
Mean wall area %	70.11 ± 0.83	0.17 (−1.23 to 1.56)	0.66 (−0.91 to 2.24)	0.413	−0.16 (−1.03 to 0.71)	0.717

The model was adjusted for age, gender, smoking, pack-years, BMI, and acute exacerbation

Each pectoralis muscle subclass was characterised in tertiles (highest tertile as reference)

*Abbreviations*: *CAT* chronic obstructive pulmonary disease assessment test, *FEV*_*1*_ forced expiratory volume in 1 s, *FVC* forced vital capacity, *mMRC* modified Medical Research Council, *PMD* pectoralis muscle density

^a^Mean ± SEM (standard error of the mean)

^b^*β*; 95% CI in parentheses (all such values)

### Combined effect of PMA and density on COPD manifestation

Combination of the two groups with the lowest PMA and PMD (tertiles 1 and 2) and the highest PMA and PMD (tertile 3) established a correlation with pulmonary function as the reference group with the highest PMA and PMD when adjusted for age, sex, smoking, pack-years, BMI, and history of acute exacerbation (Table 4). Participants with the lowest PMA and PMD exhibited significantly lower predicted FEV_1_ (*β* = − 15.42; 95% CI, − 22.56 to − 8.29), lower predicted FVC (*β* = − 8.77; 95% CI, − 15.89 to − 1.66) and severe airflow obstruction (*β* = − 0.06; 95% CI, − 0.09 to − 0.03). However, there was no statistical difference in clinically relevant traits, including mMRC and CAT scores. When comparing the CT measurement related to COPD, participants with the lowest PMA and PMD exhibited a higher EI (*β* = 4.13; 95% CI, 1.79 to 6.47), with no difference in WA% (*β* = 0.23; 95% CI, − 1.57 to 2.02). Subgroup analysis of males yielded similar results (Additional file 1: Table S3).

**Table 4:** The pulmonary outcome as determined by the combined PMA (in cm^2^) and PMD (in HU) groups in cross-sectional analyzes

	Pectoralis muscle area & density			
	Adjusted mean	Adjusted differences in mean relative to the high-high group		
	High (Tertile 3)– High (Tertile 3)^a^	High (Tertile 3)– Low (Tertile 1–2)^b^	Low (Tertile 1–2)– High (Tertile 3)^b^	Low (Tertile 1–2)– Low (Tertile 1–2)^b^
N	44	54	54	141
FVC, L	2.95 ± 0.13	−0.10 (− 0.36 to 0.16)	−0.20 (− 0.47 to 0.06)	−0.20 (− 0.43 to 0.04)
FVC, % predicted	107.85 ± 4.02	−8.23 (− 15.99 to − 0.48)	−7.16 (− 15.22 to 0.90)	−8.77 (− 15.89 to − 1.66)
FEV_1_, L	1.91 ± 0.10	−0.10 (− 0.29 to 0.08)	−0.27 (− 0.46 to − 0.08)	−0.32 (− 0.49 to − 0.15)
FEV_1_, % predicted	94.12 ± 4.03	− 9.45 (− 17.23 to − 1.67)	−12.00 (− 20.09 to − 3.92)	− 15.42 (− 22.56 to − 8.29)
FEV_1_/FVC	0.64 ± 0.02	− 0.02 (− 0.05 to 0.01)	−0.05 (− 0.08 to − 0.01)	−0.06 (− 0.09 to − 0.03)
mMRC	2.00 ± 0.23	0.29 (− 0.15 to 0.72)	0.10 (− 0.35 to 0.55)	0.11 (− 0.29 to 0.51)
CAT score	21.59 ± 1.93	3.37 (− 0.35 to 7.10)	2.58 (− 1.29 to 6.45)	2.12 (− 1.30 to 5.53)
Emphysema index	3.17 ± 1.32	3.37 (0.82 to 5.91)	2.99 (0.36 to 5.62)	4.13 (1.79 to 6.47)
Mean wall area %	70.43 ± 1.01	−0.42 (− 2.37 to 1.53)	−0.64 (− 2.66 to 1.38)	0.23 (− 1.57 to 2.02)

The model was adjusted for age, gender, smoking, pack-years, BMI and history of acute exacerbation

Each pectoralis muscle subclass (area and density) was characterised in tertiles (highest tertile as reference)

*Abbreviations*: *CAT* chronic obstructive pulmonary disease assessment test, *FEV*_*1*_ forced expiratory volume in 1 s, *FVC* forced vital capacity, *mMRC* modified Medical Research Council, *PMA* pectoralis muscle area; PMD, pectoralis muscle density

^a^Mean ± SEM (standard error of the mean)

^b^*β*; 95% CI in parentheses (all such values)

### Impact of PMA and PMD on changes in FEV_1_

The effects of baseline PMA (in cm^2^) and PMD (in HU) on FEV_1_ changes over 3-year period were assessed. During the 3-year follow-up, FEV_1_ declined by an average of 11.2 mL/year (Additional file 1: Figure S1). Both PMA and PMD were not statistically correlated with annual changes in FEV_1_; when analyzed based on sex, both PMA and PMD were not associated with changes in FEV_1_ (*P* > 0.05) (Table 5). Combination of the two groups with lower PMA and PMD (tertiles 1 and 2) and the highest PMA and PMD (tertile 3) established a correlation with the annual change in FEV_1_ as the reference group with the highest PMA and PMD when adjusted for age, sex, smoking, pack-years and BMI (Additional file 1: Table S4). There were no significant changes in FEV_1_.

**Table 5:** Adjusted differences in annual changes in FEV_1_ (mL/year) of pectoralis muscle area (PMA, in cm^2^) and density (PMD, in HU) in a longitudinal analysis

	Continuous *β* (95% CI), per 10 increase	*P*
PMA (cm^2^)		
All (*n* = 222)	6.05 (−8.81 to 20.90)	0.424
Male (*n* = 182)	6.60 (−10.72 to 23.91)	0.455
Female (*n* = 40)	21.76 (−40.35 to 83.87)	0.488
PMD (HU)		
All (*n* = 222)	−2.80 (−17.16 to 11.57)	0.703
Male (*n* = 182)	−1.34 (−21.79 to 19.11)	0.898
Female (*n* = 40)	−3.18 (− 32.32 to 25.95)	0.829

The model was adjusted for age at first visit (years), height (cm), gender, education, COPD medications use, smoking status at first visit, pack-years and time since first visit (years)

*β*; 95% CI in parentheses (all such values)

*Abbreviations*: *FEV*_*1*_ forced expiratory volume in 1 s, *FVC* forced vital capacity, *PMA* pectoralis muscle area, *PMD* pectoralis muscle density

## Discussion

The present study investigated the impact of PMD (in HU) and PMA (in cm^2^) on COPD manifestation and longitudinal changes in pulmonary function, and revealed that patients with lower PMA and PMD exhibited decreased pulmonary function and more severe emphysema, after adjusting for age, sex, smoking, pack-years, BMI, and history of acute exacerbation. Unlike PMD, PMA showed an association with CAT score. Nevertheless, both PMA and PMD did not exhibit as statistically significant association with annual changes in FEV_1_.

Skeletal muscle mass is a vital predictor of COPD-related outcomes [8, 12]. Smokers without airflow obstruction reported exhibit changes in skeletal muscle structures and muscle wasting, and low muscle mass is a prognostic factor for mortality in current smokers who do not exhibit airflow obstruction [23]. Various mechanisms induce muscle wasting, including smoking, systemic inflammation, decreased physical activity, malnutrition, and hormonal insufficiency [12, 13]. Various techniques enable the evaluation of the skeletal muscle mass, including the assessment of skin-fold thickness, bioelectrical impedance analysis (BIA), dual-energy X-ray absorptiometry (DXA), and CT [4, 24]. Despite being a reference method for assessing body composition, the use of DXA is limited because it involves additional radiation exposure, decreased accessibility, and is expensive [25]. In patients with COPD, chest CT is increasingly being used to assess disease severity, cause of acute exacerbation, and lung cancer. Without extra radiation exposure, CT can offer a comprehensive assessment of pulmonary and extrapulmonary disease, including low muscle mass in patients with COPD. Measuring the cross-sectional areas of skeletal muscle on CT could be an alternative method to assess body composition, including local skeletal muscle, which is a strong predictor of mortality in patients with COPD [11, 13]. On a single-axial CT image, PMA has been reported to be correlated with total skeletal muscle mass, as determined by BIA in healthy subjects [24]. Furthermore, CT can measure not only the muscle area but also density, reflecting the muscle quality [15]. Hence, CT is an attractive method for the comprehensive assessment of skeletal muscles in patients with COPD.

We found that lung function was low in the group with smaller PMA. BMI is correlated with an exacerbated risk for mortality and decreased pulmonary function [12, 26, 27]. After adjustment for BMI, we showed that PMA was associated with lung function independent of BMI, corroborating the results of another study [12]. A previous study examining the relationship between PMA and COPD was performed in a Caucasian population, and PMA was 29.7 cm^2^, which was larger than PMA of our study (27.0 cm^2^) [12]. This can be explained by the fact that Asian populations exhibit less expected mean skeletal muscle than Caucasian populations [28]. Although the number and extent of PMA is small compared to the Caucasian population, our study showed an association between PMA and lung function in Asians. The ratio of mid-thigh low-density muscle to the total muscle area is a predictor of muscle strength, endurance, and quality of life [15]. Low-density skeletal muscle implies lipid-rich skeletal muscle and is correlated with disability and impairment in [29]. However, no other study has investigated the correlation between PMD and lung function. The present study demonstrated that participants with lower PMD exhibited decreased lung function, whereas those with higher PMA and PMD exhibited increased lung function. While other studies only established a correlation between PMA and cross-sectional lung function, we identified a correlation between PMA and PMD and serial changes in lung function. Moreover, PMA and PMD were associated with severe airflow limitation but no longitudinal changes in FEV_1_ over a 3-year period (*P* > 0.05). Although we assessed pulmonary function changes over a - year period, future studies should aim to compare changes in CT-derived muscle area and density with pulmonary function over a more prolonged period.

Based on clinical characteristics, two types of COPD are distinguished by emphysematous (pink puffer) type with a cachectic impression and chronic bronchitis (blue bloater) type with a metabolic impression [30, 31]. Substantial differences in body composition were reported between patients with chronic bronchitis and emphysema [32]. Muscle wasting is reportedly more common in patients with emphysema [12], which induces atrophy of locomotor muscles [33]. Similarly, PMA and PMD were significantly associated with emphysema in the present study: a 10 cm^2^ decrease in PMA was associated with a 2.58% increase EI, and a 10 HU increase in PMD was significantly associated with a decrease of 2.50% in EI. Thus, we demonstrated that patients with emphysema exhibited decreased muscle mass and quality. Conversely, mean WA%, reflecting chronic bronchitis, did not exhibit a statistically significant correlation with PMA and PMD, suggesting that muscle wasting was correlated with emphysema, but not with airway disease.

To date, several studies have assessed the impact of muscle mass on the clinical manifestation of COPD, reporting that low lean body mass and fat-free mass were correlated with higher impairment in symptoms and total St George’s Respiratory Questionnaire (SGRQ) score in patients with COPD [34, 35]. Peripheral muscle changes due to the reduction in type I fibers may affect SGRQ scores [36]. Moreover, CT-derived PMA and spinal muscle area, as the respiratory muscle and antigravity muscle, are correlated with the mMRC dyspnea score and SGRQ scores, respectively [12, 13]. In this study, PMA exhibited a statistically significant correlation with CAT score, but not with the mMRC scores, which could be explained by the small smaple and the fact that most patients had mild COPD (51.5%). We identified a correlation between the pectoralis muscle and CAT scores and that PMA was significantly and inversely correlated with CAT score. PMD, however, was not associated with mMRC or CAT scores. Thus, results of this study suggest that COPD symptoms could be affected more by muscle area rather than muscle density.

This study had several limitations, the first of which was the small number of enrolled females, which precluded correlating PMA or PMD with lung function based on sex, although sex differences could exist in muscles. Diaz et al. reported that PMA was lower in females compared with males [4]. We found that PMA (males, 28.80 ± 7.72 cm^2^; females, 19.57 ± 4.84 cm^2^) and PMD (males, 45.44 ± 6.06 HU; females, 37.27 ± 9.22 HU) were higher in males compared with females. Second, PMA observed on single CT slices may vary, based on patient positioning during CT. The standard position for CT scanning is to raise the patient’s hands above their head. However, because our patients were relatively old, achieving the standard position was challenging, which may have affected the PMA results. Third, we performed manual segmentation of the pectoralis muscle. Automated programs to assess ROIs are necessary to enable more reproducible and objective analyses. Fourth, statistical power was limited due to the relatively small sample size. In addition, because only 8.2% of patients have severe GOLD grade 3 or 4 COPD, there are limitations in evaluating patient symptoms and acute exacerbations. Therefore, analysis of the model was performed by adjusting for acute exacerbation. As such, further studies are necessary, including investigations of longitudinal changes in large populations with varying COPD severity.

## Conclusions

CT-derived features of the pectoralis muscle were associated with disease severity, symptoms, and quality of life in patients with COPD, which could be helpful in determining treatment plans and estimating prognosis. Nevertheless, further research investing the effects of changes in the pectoralis muscle on longitudinal changes in lung function in large populations with COPD is warranted.

## Additional file

**Additional file 1.** Supplemental Tables and Figures: Additional Table and figures to support the findings of this study. (PDF 320 kb)

## Data Availability

The datasets used and/or analysed during the current study are available from the corresponding author on reasonable request.

## Abbreviations

BMI: Body mass index
CAT: Chronic obstructive pulmonary disease assessment test
CODA cohort: Chronic obstructive pulmonary disease in dust areas cohort
COPD: Chronic obstructive pulmonary disease
CT: Computed tomography
FEV_1_: Forced expiratory volume in 1 s
FVC: Forced vital capacity
HU: Hounsfield unit
mMRC: modified Medical Research Council
PMA: Pectoralis muscle area
PMD: Pectoralis muscle density
