# Supplementary material for: Computed tomography-derived area and density of pectoralis muscle associated disease severity and longitudinal changes in chronic obstructive pulmonary disease: a case control study
Source: Respir Res. 2019 Oct 21;20:226. doi: 10.1186/s12931-019-1191-y (PMC6805427; doi:10.1186/s12931-019-1191-y)
Supplement: Supplementary file 1 — Additional file 1. Supplemental Tables and Figures: Additional Table and figures to support the findings of this study. (PDF 320 kb) [file 12931_2019_1191_MOESM1_ESM.docx]

**Computed Tomography-derived Area and Density of Pectoralis Muscle Associated Disease Severity and Longitudinal Changes in Chronic Obstructive Pulmonary Disease: A Case Control Study**

Short title: CT-derived features of the pectoralis muscle

So Hyeon Bak,^1^ Sung Ok Kwon,^2^ Seon-Sook Han ^3^, Woo Jin Kim^3*^

^1^Department of Radiology, Kangwon National University Hospital, Kangwon National University School of Medicine, Chuncheon, Republic of Korea

^2^Biomedical Research Institute, Kangwon National University Hospital, Chuncheon, Republic of Korea

^3^Department of Internal Medicine and Environmental Health Center, School of Medicine, Kangwon National University, Chuncheon, Republic of Korea

*Correspondence to:

Woo Jin Kim

Department of Internal Medicine, School of Medicine, Kangwon National University, Chuncheon, Republic of Korea

1 Kangwondaehak-gil, Chuncheon, Gangwon-do, 24341, Republic of Korea

Tel.: +82-32-258-9364

Fax: +82-32-258-2404

E-mail: [pulmo2@kangwon.ac.kr](mailto:pulmo2@kangwon.ac.kr)

**Table S1.** The pulmonary outcome as determined by the pectoralis muscle area (PMA, in cm2) among male in a cross-sectional analysis.

|  | Adjusted mean | Adjusted difference in mean relative to tertile 3 | |  |  |  |
| --- | --- | --- | --- | --- | --- | --- |
|  | Tertile 3 (highest)^*^ | Tertile 2^†^ | Tertile 1 (lowest) | *P*-trend | Continuous *β* (95% CI), per 10 cm^2^ increase | *P* |
| *N* | 97 | 87 | 52 |  |  |  |
| PMA (cm^2^), median (min–max) | 34.20 (29.83–56.42) | 26.63 (23.44 to 29.82) | 19.34 (8.31–23.44) |  |  |  |
| FVC, L | 3.35 ± 0.12 | −0.10 (−0.30 to 0.10) | −0.28 (−0.54 to −0.01) | 0.046 | 0.17 (−0.04 to 0.31) | 0.013 |
| FVC, % predicted | 97.91 ± 3.49 | −2.6 (−8.30 to 3.09) | −8.45 (−15.96 to −0.94) | 0.033 | 4.56 (0.67 to 8.45) | 0.022 |
| FEV_1_, L | 2.06 ± 0.09 | −0.21 (−0.36 to −0.06) | −0.34 (−0.54 to −0.15) | <0.001 | 0.22 (0.12 to 0.32) | <0.001 |
| FEV_1_, % predicted | 82.51 ± 3.55 | −7.64 (−13.43 to−1.86) | −13.73 (−21.36 to −6.11) | <0.001 | 8.14 (4.22 to 12.07) | <0.001 |
| FEV_1_/FVC | 0.61 ± 0.02 | −0.04 (−0.06 to −0.01) | −0.06 (−0.10 to −0.03) | <0.001 | 0.04 (0.02 to 0.05) | <0.001 |
| mMRC | 1.96 ± 0.19 | −0.16 (−0.47 to 0.16) | 0.17 (−0.25 to 0.58) | 0.616 | −0.12 (−0.34 to 0.10) | 0.270 |
| CAT score | 22.35 ± 1.70 | −0.8 (−3.57 to 1.97) | 3.32 (−0.33 to 6.97) | 0.145 | −1.30 (−3.21 to −0.61) | 0.181 |
| Emphysema index | 7.21 ± 1.27 | 1.09 (−1.00 to 3.18) | 4.04 (1.27 to 6.82) | 0.007 | −2.37 (−3.81 to −−0.92) | 0.001 |
| Mean wall area % | 69.51 ± 0.89 | 0.14 (−1.31 to 1.60) | 0.14 (−1.80 to 2.07) | 0.871 | −0.29 (−1.30 to 0.72) | 0.571 |

The model was adjusted for age, smoking, pack-years, BMI and history of acute exacerbation.

Each pectoralis muscle subclass (area and density) was characterised in tertiles (highest tertile as reference).

*Mean ± SEM (standard error of the mean).

^†^*β*; 95% CI in parentheses (all such values).

*Definition of abbreviations*: CAT, chronic obstructive pulmonary disease assessment test; FEV_1_, forced expiratory volume in 1 s; FVC, forced vital capacity; mMRC, modified Medical Research Council; PMA, pectoralis muscle area

**Table S2.** The pulmonary outcome as determined by the pectoralis muscle density (PMD, in HU) among male in a cross-sectional analysis.

|  | Adjusted mean | Adjusted difference in mean relative to tertile 3 | |  |  |  | |
| --- | --- | --- | --- | --- | --- | --- | --- |
|  | Tertile 3 (highest)^*^ | Tertile 2^†^ | Tertile 1 (lowest) | *P*_trend_ | Continuous *β* (95% CI), per 10 HU increase | | *P* |
| N | 94 | 86 | 56 |  |  | |  |
| PMD, median (min-max) | 50.66 (47.80–58.88) | 44.90 (41.65 to 47.73) | 37.60 (10.33–41.55) |  |  | |  |
| FVC, L | 3.28 ± 0.12 | −0.05 (−0.25 to 0.15) | −0.06 (−0.29 to 0.18) | 0.615 | 0.08 (−0.08 to 0.23) | | 0.324 |
| FVC, % predicted | 98.19 ± 3.43 | −4.18 (−9.83 to 1.46) | −6.36 (−12.94 to 0.22) | 0.050 | 4.53 (0.28 to 8.79) | | 0.037 |
| FEV_1_, L | 1.96 ± 0.09 | −0.09 (−0.24 to 0.06) | −0.13 (−0.31 to 0.04) | 0.119 | 0.16 (0.05 to 0.27) | | 0.006 |
| FEV_1_, % predicted | 81.04 ± 3.51 | −5.96 (−11.74 to −0.19) | −9.94 (−16.67 to −3.20) | 0.003 | 8.65 (4.36 to 12.94) | | <0.001 |
| FEV_1_/FVC | 0.59 ± 0.02 | −0.02 (−0.04 to 0.01) | −0.03 (−0.06 to −0.00) | 0.036 | 0.04 (0.02 to 0.05) | | <0.001 |
| mMRC | 1.82 ± 0.19 | 0.15 ( −0.17 to 0.46) | 0.19 (−0.18 to 0.56) | 0.280 | −0.19 (−0.42 to 0.05) | | 0.118 |
| CAT score | 21.73 ± 1.68 | 1.13 (−1.64 to 3.89) | 2.17 (−1.05 to 5.39) | 0.180 | −2.08 (−4.16 to −0.01) | | 0.049 |
| Emphysema index | 6.96 ± 1.25 | 2.36 (0.28 to 4.43) | 2.67 (0.24 to 5.11) | 0.021 | −2.96 (−4.5 to -1.43) | | <0.001 |
| Mean wall area % | 69.42 ± 0.87 | 0.16 (−1.28 to 1.59) | 0.48 (−1.21 to 2.16) | 0.586 | −0.25 (−1.34 to 0.83) | | 0.644 |

The model was adjusted for age, smoking, pack-years, BMI and history of acute exacerbation.

Each pectoralis muscle subclass (area and density) was characterised in tertiles (highest tertile as reference).

*Mean ± SEM (standard error of the mean).

^†^*β*; 95% CI in parentheses (all such values).

*Definition of abbreviations*: CAT, chronic obstructive pulmonary disease assessment test; FEV_1_, forced expiratory volume in 1 s; FVC, forced vital capacity; mMRC, modified Medical Research Council; PMD, pectoralis muscle density

**Table S3.** The pulmonary outcome as determined by the combined PMA (in cm2) and PMD (in HU) groups among male in a cross-sectional analysis.

|  | Pectoralis muscle area & density | | | |
| --- | --- | --- | --- | --- |
|  | Adjusted mean | Adjusted differences in mean relative to the high-high group | | |
|  | High (Tertile 3)–  High (Tertile 3)^*^ | High (Tertile 3)–  Low (Tertile 1–2)^†^ | Low (Tertile 1–2)–  High (Tertile 3) | Low (Tertile 1–2)–  Low (Tertile 1–2) |
| N | 44 | 53 | 50 | 89 |
| FVC, L | 3.38 ± 0.14 | -0.09 ( -0.37 to 0.18) | -0.21 ( -0.49 to 0.08) | -0.18 ( -0.43 to 0.07) |
| FVC, % predicted | 101.26 ± 3.93 | -7.78 ( -15.37 to -0.18) | -6.68 ( -14.76 to 1.39) | -8.41 ( -15.46 to -1.36) |
| FEV_1_, L | 2.10 ± 0.10 | -0.10 ( -0.30 to 0.10) | -0.26 ( -0.47 to -0.05) | -0.3 ( -0.48 to -0.12) |
| FEV_1_, % predicted | 86.49 ± 3.98 | -8.99 ( -16.67 to -1.30) | -11.09 ( -19.26 to -2.92) | -14.66 ( -21.8 to -7.53) |
| FEV_1_/FVC | 0.61 ± 0.02 | -0.02 ( -0.05 to 0.02) | -0.04 ( -0.08 to -0.01) | -0.06 ( -0.09 to -0.02) |
| mMRC | 1.83 ± 0.22 | 0.31 ( -0.11 to 0.73) | 0.02 ( -0.43 to 0.47) | 0.10 ( -0.29 to 0.49) |
| CAT score | 20.94 ± 1.93 | 3.59 ( -0.14 to 7.31) | 2.06 ( -1.9 to 6.02) | 1.94 ( -1.51 to 5.40) |
| Emphysema index | 5.72 ± 1.43 | 3.53 (0.75 to 6.31) | 2.68 ( -0.25 to 5.62) | 3.89 (1.30 to 6.47) |
| Mean wall area % | 69.63 ± 1.00 | -0.35 ( -2.28 to 1.59) | -0.54 ( -2.59 to 1.51) | 0.19 ( -1.61 to 2.00) |

The model was adjusted for age, smoking, pack-years, BMI and history of acute exacerbation

Each pectoralis muscle subclass (area and density) was characterised in tertiles (highest tertile as reference).

Bolded data are statistically significant.

*Mean ± SEM (standard error of the mean).

^†^*β*; 95% CI in parentheses (all such values).

*Definition of abbreviations*: CAT, chronic obstructive pulmonary disease assessment test; FEV_1_, forced expiratory volume in 1 s; FVC, forced vital capacity; mMRC, modified Medical Research Council; PMA, pectoralis muscle area; PMD, pectoralis muscle density

**Table S4.** Adjusted differences in annual changes in FEV_1_ (mL/year) for combined groups of the pectoralis muscle area (PMA) and density (PMD) in longitudinal analysis

|  | Adjusted mean annual change in FEV_1_ | Adjusted difference in mean annual change in FEV_1_ relative to Tertile 1 | | | |
| --- | --- | --- | --- | --- | --- |
|  | high (Tertile 3) –  High (Tertile 3) | | High (Tertile 3) –  Low (Tertile 1-2) | Low (Tertile 1-2) –  High (Tertile 3) | Low (Tertile 1-2) –  Low (Tertile 1-2) |
| N | 30 | | 43 | 45 | 104 |
| All (n=222) | 7.45 (−27.21 to 42.11) | | −8.00 (−52.70 to 36.70) | −31.98 (−76.39 to 12.43) | -21.07 (−60.00 to 17.87) |
| Male (n=182) | −5.47 (-42.11 to 31.17) | | 5.99 (−42.14 to 54.11) | −9.32 (−56.98 to 38.33) | −9.93 (−51.25 to 38.33) |
| Female (n=40) | 88.37 (−12.59 to 189.34) | | −89.99 (−209.31 to 29.33) | −151.65 (−271.20 to -32.10) | −93.45 (−203.84 to 16.94) |

Model was adjusted for age at first visit (years), height (cm), gender, education, COPD medications use (LAMA/LABA/SABA/ICS/LABA), smoking status at first visit, pack-years of smoking, and time since first visit (years).

β; 95% CI in parentheses (all such values).

*Definition of abbreviations*: FEV_1_: forced expiratory volume in 1 s

**figure S1.** Distribution of estimated annual rates of change in FEV_1_ over 3-year period in subjects with COPD (n=222, average = -11.2 mL/year)

*Definition of abbreviations*: FEV_1_: forced expiratory volume in 1 s
